# Side effects of intraoral devices for OSAS treatment^☆^

**DOI:** 10.1016/j.bjorl.2017.09.003

**Published:** 2017-10-14

**Authors:** Andressa Otranto de Britto Teixeira, Ana Luiza Ladeia Andrade, Rhita Cristina da Cunha Almeida, Marco Antonio de Oliveira Almeida

**Affiliations:** a Universidade do Estado do Rio de Janeiro (Uerj), Faculdade de Odontologia, Ortodontia, Rio de Janeiro, RJ, Brazil; b North Carolina State University, Chapel Hill, United States

**Keywords:** Obstructive sleep apnea syndrome, Mandibular advancement, Continuous positive airway pressure, Síndrome da apneia obstrutiva do sono, Avanço mandibular, Pressão positiva contínua nas vias aéreas

## Abstract

**Introduction:**

Intraoral devices have increasingly assumed a key role in the treatment of obstructive sleep apnea syndrome, but there are limitations to their indication and side effects that result from their continuous use, as well as the use of the continuous positive airway pressure device.

**Objectives:**

To evaluate the changes in dental positioning caused by the continuous use of mandibular advancement devices.

**Methods:**

A prospective longitudinal study with a sample of 15 patients, with evaluation of complete documentation after a mean time of 6.47 months, assessed changes in dental positioning due to the use of the Twin Block oral device for the treatment of patients with apnea. The following variables were evaluated: overjet, overbite, upper and lower intermolar distances, upper and lower intercanine distances, Little's irregularity index and the incisor mandibular plane angle. An intraclass correlation test was performed and a correlation index > 0.08 was accepted. After verifying the normal sample distribution (Shapiro-Wilks), a parametric test was used (*t* test), with a significance level set at 5%.

**Results:**

There was a decrease in the values of overjet, overbite and Little's irregularity index, whereas there was an increase in the lower intercanine distance and IMPA values. All these variables are influenced, at different levels, by the forward inclination of the lower incisors, an action that can be expected due to the force applied by the device on the dentition. The other variables did not show statistically significant differences.

**Conclusion:**

After a mean time of 6.47 months of use of the mandibular advancement device, there were statistically significant changes in the dental positioning, but they were not clinically relevant. However, it is relevant that this device is commonly in use over long periods of time, making the monitoring of these patients of the utmost importance for the duration of their therapy.

## Resumo

**Introdução:**

Os aparelhos intraorais têm assumido cada vez mais um papel importante no tratamento da síndrome da apneia obstrutiva do sono, mas existem limitações a sua indicação e efeitos colaterais com o seu uso contínuo, assim como com o uso do aparelho de pressão aérea positiva contínua.

**Objetivos:**

Avaliar as alterações no posicionamento dentário produzido pelo uso contínuo do aparelho de projeção mandibular.

**Método:**

Através de estudo longitudinal prospectivo com amostra de 15 pacientes, com avaliação de documentações completas após um tempo médio 6,47 meses do uso do aparelho oral de *Twin Block* para tratamento de pacientes com apneia, foram avaliadas as alterações do posicionamento dos dentes decorrentes do seu uso. As seguintes variáveis foram avaliadas: *overjet*, *overbite*, distâncias intermolares superior e inferior, distâncias intercaninos superior e inferior, índice de irregularidade de Little e ângulo do plano incisivo mandibular. Foi feito teste de correlação intraclasse e foram aceitos índices de correlação acima de 0,08. Após atestada a distribuição normal da amostra (Shapiro-Wilks), foi usado um teste paramétrico (teste *t*), com nível de significância de 5%.

**Resultados:**

Houve diminuição nos valores de *overjet, overbite* e irregularidade de Little e aumento nos valores da distância intercanino inferior e do ângulo do plano incisivo mandibular. Todas essas variáveis sofrem influência, com diferentes expressividades, da inclinação para frente dos incisivos inferiores, uma ação que pode ser esperada devido à força aplicada pelo aparelho sobre a dentição. As demais variáveis não demostraram diferenças estatisticamente significativas.

**Conclusão:**

Houve mudanças estatisticamente significativas no posicionamento dos dentes, porém clinicamente sem relevância, com um tempo médio de uso de 6,47 meses do aparelho de avanço mandibular. Contudo, deve-se considerar que o uso desta aparelhagem, é comum durante longos períodos, fazendo com que seja de suma importância o acompanhamento desses pacientes a longo prazo.

## Introduction

Obstructive sleep apnea syndrome (OSAS) is characterized by recurrent events of airway obstruction during sleep, resulting in micro-awakenings that interfere with normal sleep architecture.^1^, ^2^ This syndrome has received much attention because of its high degree of morbidity. Increased number of traffic and work-related accidents^3^ are associated with it, as well as difficult-to-control arterial hypertension and pulmonary hypertension.^4^, ^5^, ^6^ Several types of treatment have been applied to patients aiming to control OSAS. These measures range from sleep hygiene methods^7^, ^8^ and body weight reduction to surgical treatments,^9^ such as maxillo-mandibular advancements, nasal surgeries and tracheostomies.^10^ However, the most commonly used methods are those aimed at the clinical control of the obstructive phenomena.^11^ Among the most common are the Continuous Positive Airway Pressure (CPAP) and intraoral devices.^12^, ^13^, ^14^ There is a great diversity of intraoral devices available in the market that are used to treat OSAS.^13^ There are devices that pull the tongue musculature into a bulb (TRD - Tongue Retainer Device), plastic devices sold directly to patients without any individualization, and several types of individualized mandibular advancement devices. The most commonly used intraoral devices that show greater efficiency, are those that provide individualized mandibular advancement taking with it all the suprahyoid muscles, promoting airway widening, mainly in the oropharyngeal region.^15^, ^16^ The Twin Block is one of those devices that promote individualized mandibular advancement, and attains promising results in the treatment of OSAS.^2^ Although the CPAP is considered the gold standard treatment for OSAS,^17^, ^18^ intraoral devices can already be considered the first-choice treatment for cases of primary snoring, UARS (Upper Airway Resistance Syndrome), mild to moderate OSAS, as well as being a secondary management strategy for patients with severe OSAS who do not adhere to CPAP treatment, because many find CPAP uncomfortable. This makes adherence to oral devices higher than to CPAP.^13^, ^18^, ^19^ Recent studies have demonstrated that although patients generally have less reduction in the indeces that measure disease severity, such as the Apnea and Hypopnea Index (AHI) per hour of sleep, the reduction in blood pressure is similar with the intraoral device compared to CPAP, and there is no difference in the prevention of deaths from cardiovascular diseases caused by OSAS.^9^, ^20^ Intraoral devices are increasingly assuming a more important role in the treatment of this syndrome, but there are limitations to their indications and side effects that occur with their continuous use, just as there are with the use of CPAP.^18^, ^21^, ^22^ The main limitation regarding the indication of mandibular advancement devices is the need for a minimum number of teeth in good condition for device fixation.^2^, ^15^ With respect to the side effects, much atention is given to those related to CPAP, such as difficult adherence, nasal congestion, airway dryness, it seems that less attention is given to the side effects produced by intraoral devices.^23^, ^24^, ^25^, ^26^ This study assessed the changes in dental positioning resulting from the use of a mandibular advancement device, an effect that may impair patient dental occlusion and eventually lead to the discontinuation of this type of treatment.

## Method

**Figure 1:**
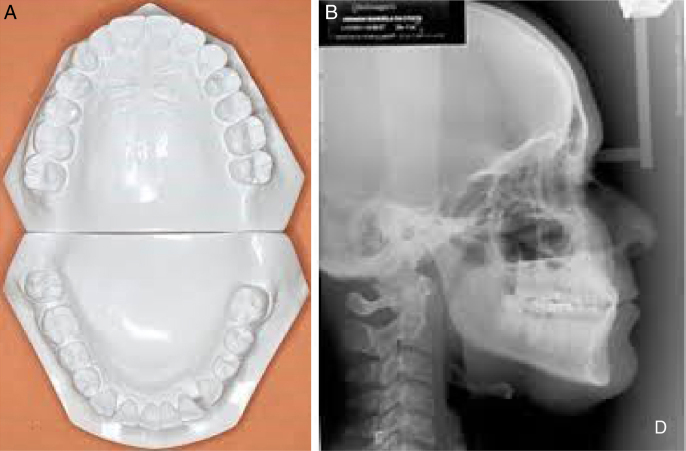
**A**, Study models used for assessment. These models were scanned so that the digital measurements could be performed. B, Radiographs used to assess the position of the incisors.

**Figure 2:**
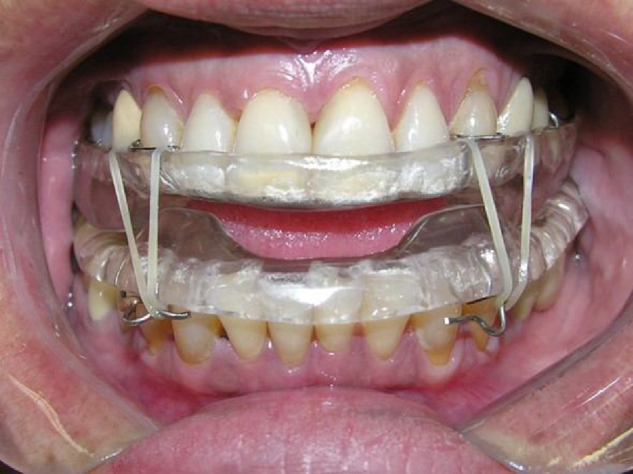
Twin Block Device.

This is a longitudinal prospective study, which assessed complete documentation (Fig. 1A and 1B), consisting of lateral cephalometric radiographs and initial and final cephalometric models (T1 and T2 respectively), after a mean time of 6.47 months of use of the oral device for the treatment of patients with mild to moderate OSAS, using a Twin Block mandibular protraction device^27^ (Fig. 2). Cephalometric models and radiographs of patients who had all the necessary teeth (upper and lower first molars, upper and lower canines and the four lower incisors) were included in the sample to reproduce the measures that were assessed in this study.

Fifteen patients who met the inclusion criteria were included in this study sample (Table 1) and their documentation was obtained and later assessed.

**Table 1:** Values of Apnea and Hypopnea Index (AHI), Body Mass Index (BMI), age and gender of the patients in the sample.

**Patient**	**AHI**	**BMI**	**Age**	**Gender**
1	22.3	25.8	34.5	Female
2	28.1	33.4	42.4	Male
3	14.4	38.1	66.3	Male
4	26.6	29.6	35.2	Male
5	12.5	29.4	39.4	Male
6	26.3	36.2	58.6	Female
7	20.8	24.5	33.2	Male
8	31.6	38.4	46.2	Female
9	25.1	36.3	48.4	Female
10	12.9	30.7	55.6	Male
11	13.8	32.4	51.4	Male
12	20.2	29.0	52.8	Female
13	27.4	34.2	57.3	Male
14	22.1	28.8	50.0	Female
15	27.3	24.4	37.2	Male
Mean	22.09	31.41	47.23	-
Standard Deviation	5.99	4.49	9.70	-

**Figure 3:**
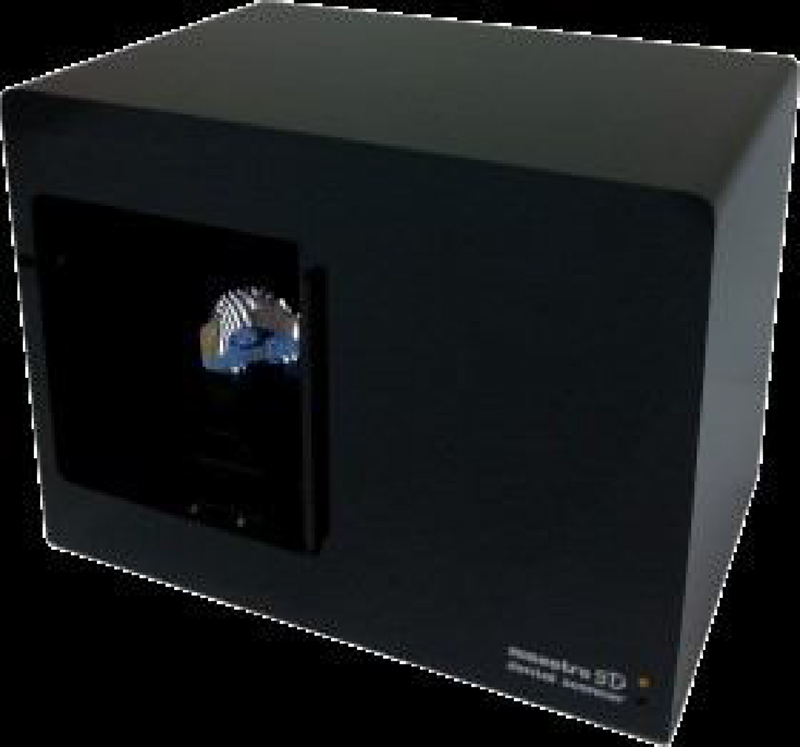
Maestro Scanner 3D image.

**Figure 4:**
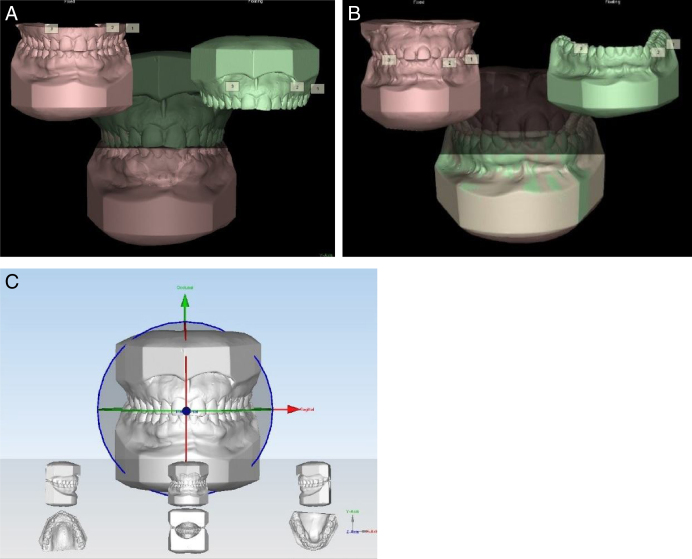
A, Overlapping of the models using the Maestro 3D Easy Dental Scan program (Upper Model); B, Overlapping of the models using the Maestro 3D Easy Dental Scan program (Lower Model); and C, Definition of the sagittal, occlusal and transverse planes (X, Y and Z) using the Maestro 3D Ortho Studio program.

The models were scanned using the Maestro 3D scanner (Fig. 3), with acquisition technology using the structured light projection technique, acquisition speed of thousands of points in a few seconds, accuracy of 10 microns and resolution of 0.07 mm, using the Maestro 3D Easy Dental Scan program, through which the upper, lower and occlusion models were digitized and then superimposed, creating 3 “floating points” in the upper and lower models and 3 “fixed points” in the occlusion models, where they should be in the same position in both, so there would be a better alignment between these models (Fig. 4A and 4B). Afterwards, they were imported into the 3D Maestro Ortho Studio program for the manipulation and definition of the sagittal, occlusal and transverse planes, represented by the X, Y and Z planes, respectively, of each digital model pair in occlusion (Fig. 4C) and then exported to the Geomagic Qualify 2013, where measurements of the digital models were reproduced.

The cephalometric radiographs were scanned in an HP Scanjet 4890 scanner and traced by the Dolphin Imaging software through an analysis specifically designed to reproduce the measurement that would be assessed.

**Figure 5:**
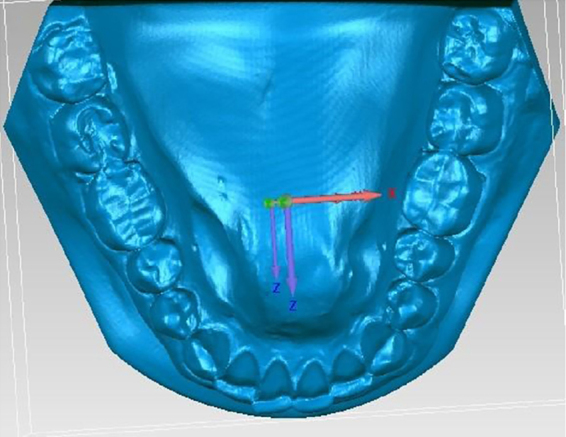
Visualization of intercanine and intermolar distance measurement.
- Upper and lower intermolar distance (UIMD and LIMD respectively): measured from the one central fossa to another central fossa of the first molars in plane X in millimeters in both arches (Fig. 5);
- Anteroinferior dental crowding: it will be assessed based on the analysis of Little's Irregularity^28^, ^29^, ^30^ in the lower arch, through the summation in millimeters of the contact points from one canine mesial surface to another, represented by the letters A, B, C, D and E (Fig. 6A and B);

The measurements obtained from the scanned models before and after treatment with the Twin Block device were reproduced, in order to determine any intra- and inter-arch changes, such as:

- Upper and lower intercanine distance (UICD and LICD respectively): measured from one tip to the other tip of the canine cusp in millimeters (mm) in the X plane in both arches (Fig. 5);

**Figure 6:**
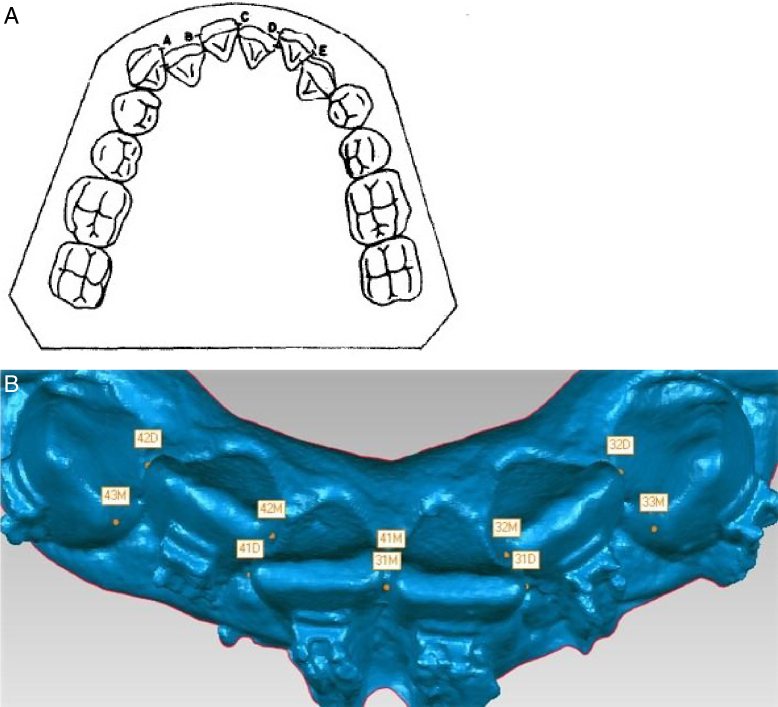
A, Drawing representing the measurement of the distances to calculate Little's Irregularity index; B, Measurement performed on the digital model.
- Horizontal distance between the upper anterior teeth and the lower anterior teeth (overjet): measured from the most vestibular incisal border of the upper incisor to the incisal border of the lower incisor in millimeters, horizontally, represented by the Z plane (Fig. 7);

**Figure 7:**
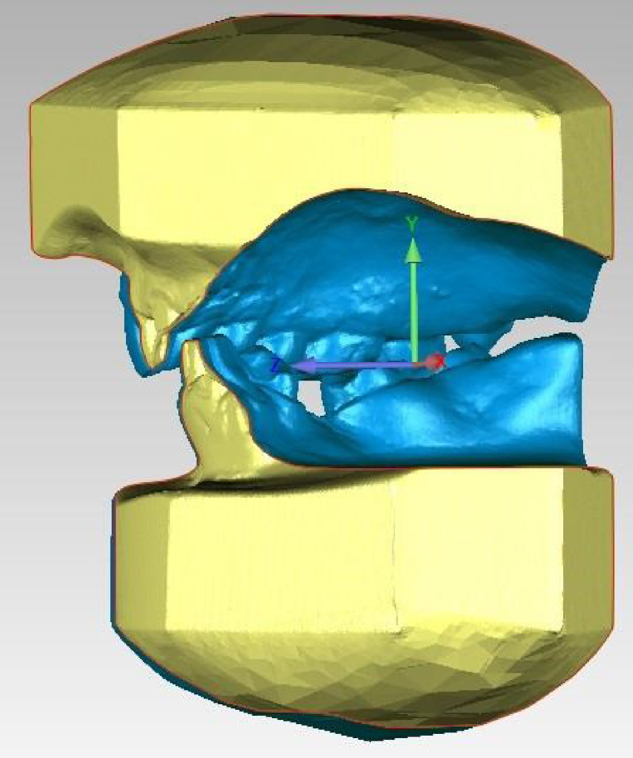
Measurement of Overjet (Plane Z) and Overbite (Plane Y) on digital model.
- Amount of overlapping of anteroinferior teeth by the anterosuperior teeth (overbite): measured from the amount of vertical overlap of the incisal border of the central upper incisor to the incisal border of the more extruded lower central incisor in millimeters, vertically,^31^ represented by plane Y (Fig. 7);
- The angulation of the lower incisors was assessed in the lateral cephalometric radiographs taken before and after treatment, which in the case of this study, were analyzed through Tweed's IMPA angle,^32^, ^33^ consisting of the mandibular plane, traced using as reference a plane tangential to the lower border of the mandible and the long axis of the lower incisor.

The variables were measured twice, in 10 pairs of cephalometric models and 10 cephalometric radiographs (5 pairs of models and 5 radiographs at time T1 and the other half at time T2, corresponding to 30% of the sample) randomly selected by the same evaluator with a minimum interval of two weeks, with the results between the measures being compared using the intraclass correlation test; a correlation index > 0.08 was accepted.

The software Statistica 7.0 was used to perform the statistical tests. The Shapiro-Wilks Test - *W* test was used to verify the normality of the sample data. The interpretation of the observed variable values of overjet, overbite, UIMD, LIMD, UICD, LICD, Little's Irregularity and IMPA was performed with a decision level of α = 0.05. After verifying the normal distribution of the sample, a parametric test (*t* test), with a significance level of 5%, was used to compare the several parameters at times T1 and T2.

The research project was registered in the Brazil Platform and submitted to the Research Ethics Committee for approval, obtaining a favorable opinion (CAAE n° 34300714.0.0000.5259). All patients signed the Free and Informed Consent Form (FICF) for inclusion in the study.

## Results

The patients included in the sample underwent analysis of their documentation, cephalometric models and lateral radiographs at the initial moment (T1) and after 6.47 months (SD = 2.01) of use of the Twin Block device (T2). The measurements were repeated after a two-week interval, and good reproducibility of these variables was verified (Table 2, Table 3).

**Table 2:** Results of the *t*-Test for paired samples of before and after type, for the variable method error with a two-week interval on T1.

**t-Test for paired samples**	**Mean of difference**	**Standard deviation of difference**	***t***	**Degrees of freedom**	***p*****-value**
Overjet (mm)	0.45	0.442	2.275	4	0.085
Overbite (mm)	1.73	1.658	2.43	4	0.079
UIMD (mm)	0.58	0.616	2.106	4	0.103
LIMD (mm)	0.086	0,337	0.571	4	0.599
UICD (mm)	–0.256	0.391	–1.463	4	0.217
LICD (mm)	–0.106	0.288	–0.822	4	0.457
Little's Irregularity (mm)	0.036	1.153	0.069	4	0.947
IMPA (degrees)	–0.68	0.858	–1.771	4	0.151

**Table 3:** Results of the *t*-Test for paired samples of before and after type, for the variable method error with a two-week interval on T2.

**t-Test for paired samples**	**Mean of difference**	**Standard deviation of difference**	***t***	**Degrees of freedom**	***p*****-value**
Overjet (mm)	–0.256	0.284	–2.013	4	0.1143
Overbite (mm)	–0.010	0.387	–0.058	4	0.957
UIMD (mm)	–0.078	0.380	–0.458	4	0.670
LIMD (mm)	–0.006	0.254	–0.053	4	0.960
UICD (mm)	0.152	0.364	0.932	4	0.404
LICD (mm)	0.030	0.367	0.183	4	0.864
Little's irregularity (mm)	–0.010	0.225	–0.099	4	0.925
IMPA (degrees)	0.940	2.007	1.047	4	0.354

The Shapiro-Wilks test - W test confirmed the normality of the sample (*p* > 0.05) (Table 4).

**Table 4:** Results of the Shapiro-Wilks normality test, W test, for the variables under study.

**Variable**	**Sample size**	**Mean**	**Standard Deviation**	***W***	***p***
Overjet	15	3.70	1.98	0.91931	0.188
Overbite	15	2.95	1.62	0.94583	0.4613
UIMD	15	48.53	3.38	0.92433	0.2242
LIMD	15	44.99	3.10	0.93999	0.3823
UICD	15	33.86	1.93	0.97134	0.8774
LICD	15	26.18	2.18	0.98258	0.9841
Little's Irregularity	15	5.59	4.69	0.91218	0.1462
IMPA	15	95.23	8.50	0.90667	0.1204

**Figure 8:**
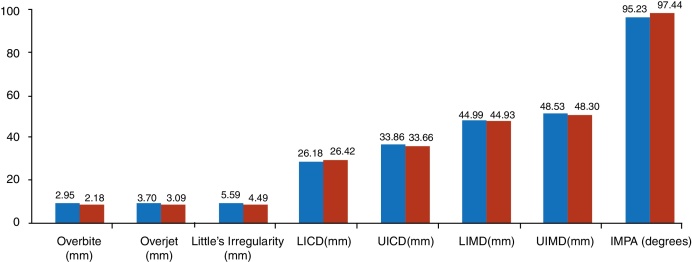
Comparison between the means of the variables observed at times T1 and T2.

The pairs were then compared using Student's *t*-test to verify differences in dental positioning between the two times (T1 and T2) due to the use of the mandibular advancement device. The variables that showed statistically significant differences were overjet, overbite, LICD, Little's Irregularity and IMPA (Table 5 and Fig. 8). There was a decrease in the overjet, overbite and Little's Irregularity values and an increase in the values of LICD and IMPA. All these variables are influenced, at different levels, by the forward inclination of the lower incisors, an action that can be expected due to the force applied by the device on the dentition. The other variables (UICD, UIMD, LIMD) did not show any statistically significant differences.

**Table 5:** Results of the *t*-test for paired samples, of before and after type.

***t*****-Test for paired samples**	**Mean of difference**	**Standard deviation of difference**	***t***	**Degrees of freedom**	***p*****-value**
Overjet (mm) (T1 × T2)	0.61	0.457	5.172	14	< 0.001
Overbite (mm) (T1 × T2)	0.76	1.167	2.537	14	0.023
UIMD (mm) (T1 × T2)	0.23	0.525	1.731	14	0.105
LIMD (mm) (T1 × T2)	0.056	0.587	0.369	14	0.717
UICD (mm) (T1 × T2)	0.195	0.638	1.184	14	0.255
LICD (mm) (T1 × T2)	–0.238	0.187	–4.917	14	< 0.001
Little's Irregularity (mm) (T1 × T2)	1.10	1.370	3.109	14	0.007
IMPA (degrees) (T1 × T2)	–2.206	1.81	–4.541	14	< 0.001

## Discussion

Awareness of the appropriate indications for the use of intraoral devices to treat OSAS is of utmost importance so they can be utilized when it will be most beneficial to the patient.

The short-term side effects caused by the devices are already well known.^34^, ^35^, ^36^, ^37^ The medium and long-term effects still need further studies and greater understanding so they can be diagnosed and treated,^38^, ^39^, ^40^ which is very important, since any clinical intervention for the treatment of OSAS should be considered a long-term therapy.

This study aimed to evaluate the dental changes produced by a device used in the treatment of OSAS that advances the mandible. Statistically significant alterations were observed in overjet, overbite, LICD, Little's Irregularity and IMPA, all of which can be explained by the projection of the lower incisors, probably occurring due to the projection force applied to the lower arch by the device. These data are in agreement with the studies of Marklund et al.,^41^ who found changes in overjet of -0.4 mm ± 0.8 mm and of -0.4 mm ± 0.7 mm in overbite, in agreement with the results of Hammond et al.,^42^ who found statistically significant changes in the anterior movement of lower incisors of 0.5 ± 0.12 mm, even though, according to these authors, they were not clinically significant, also similar to those of Roberton, Herbison and Harkness,^43^ who also observed these changes in overjet and overbite, and the study of Martinez et al.,^25^ who observed changes in incisor inclination, in addition to molar position alterations, similar to those found in our study.

An important factor in dental modifications may be the amount of projection caused by these devices. Marklund et al.^41^ concluded that the orthodontic side effects obtained during treatment with an intraoral device for snoring and apnea syndrome are small, especially if the advancement of this mandibular device is less than 6.0 mm. In this study, the mandibular advancement achieved in the patients ranged from 5.0 to 8.0 mm, and in most patients this advancement was greater than 6.0 mm. It is worth mentioning that the best results, considering the reduction in AHI, are found when an advancement ≥ 75% of maximal protrusive capacity is achieved, which often exceeds 6.0 mm.

Another important issue may be the time of follow-up,^23^, ^38^, ^39^, ^40^ as these changes seem to be progressive. In our study, although the statistical data showed values with clinically significant differences, they might not be relevant, since the differences between T1 and T2 in these variables were small. This fact may be related to the time of follow-up of 6.47 months. However, in the studies by Marklund, Franklin and Persson^41^ and Marklund,^40^ for instance, with a time of follow-up of approximately 2.5 ± 0.5 years and 5.4 ± 0.8 years respectively, different from the length of time evaluated in this study, the results were shown to be similar. The same was also observed by Fransson et al.^36^ who followed 65 patients using the mandibular advancement device for two years. In the studies carried out by Almeida et al.,^39^, ^44^ which assessed the use of the mandibular advancement device for the treatment of OSAS with a long-term evaluation, on average 7.4 years, with 70 patients having their plaster models of the dental arches and cephalometric radiographs visually compared, it was observed that changes occurred in 85.7% of the cases. Similar results were found by Robertson,^38^ who demonstrated that the most significant dental changes occurred at the 30-month follow-up, stating that dental and skeletal changes may be progressive over time. He then recommended that all patients should be informed of the potential of these changes prior to treatment and be followed by a dentist throughout treatment. The same recommendation was given by Clark, Sohn and Hong^35^ and by Perez et al.,^45^ who also observed that 26% of users of mandibular advancement devices experienced a painless, but irreversible change in their occlusions.

These changes must be followed closely by a dentist, as they occur most of the time without the patients noticing it. Almeida et al.,^26^ based on a literature review aimed at answering the main doubts regarding the use of intraoral devices for the treatment of OSAS, concluded that the main occlusal side effects were overjet reduction, overbite reduction, proclination of the lower incisors, and establishment of a lateral open bite, although most of the times without generating great discomfort to the patients, in agreement with Perez et al.^45^

## Conclusions

There were statistically significant changes in overjet, overbite, LICD, Little's Irregularity and IMPA, but these were not clinically relevant, after a mean time of use of 6.47 months; however, it must be considered that these devices are commonly used for long periods of time, making it very important to follow these patients for the duration of treatment.

## Conflicts of interest

The authors declare no conflicts of interest.
